# MEG Microstates: An Investigation of Underlying Brain Sources and Potential Neurophysiological Processes

**DOI:** 10.1007/s10548-024-01073-z

**Published:** 2024-08-08

**Authors:** Christian Valt, Angelantonio Tavella, Cristina Berchio, Dylan Seebold, Leonardo Sportelli, Antonio Rampino, Dean F. Salisbury, Alessandro Bertolino, Giulio Pergola

**Affiliations:** 1 Department of Translational Biomedicine and Neuroscience, University of Bari Aldo Moro, Bari, Italy; 2 Department of Mental Health, ASL Bari, Bari, Italy; 3 Clinical Neurophysiology Research Laboratory, Western Psychiatric Hospital, University of Pittsburgh School of Medicine, Pittsburgh, PA USA; 4 Lieber Institute for Brain Development, Johns Hopkins Medical Campus, Baltimore, MD USA; 5 Psychiatric Unit, Bari University Hospital, Bari, Italy; 6 Department of Psychiatry and Behavioral Sciences, Johns Hopkins University School of Medicine, Baltimore, MD USA

**Keywords:** Resting-state microstates, Magnetoencephalography, Mismatch negativity, Occipital alpha activity, Source-reconstructed brain activation

## Abstract

Microstates are transient scalp configurations of brain activity measured by electroencephalography (EEG). The application of microstate analysis in magnetoencephalography (MEG) data remains challenging. In one MEG dataset (*N* = 113), we aimed to identify MEG microstates at rest, explore their brain sources, and relate them to changes in brain activity during open-eyes (ROE) or closed-eyes resting state (RCE) and an auditory Mismatch Negativity (MMN) task. In another dataset of simultaneously recorded EEG-MEG data (*N* = 21), we investigated the association between MEG and EEG microstates. Six MEG microstates (mMS) provided the best clustering of resting-state activity, each linked to different brain sources: mMS 1–2: left/right occipito-parietal; mMS 3: fronto-temporal; mMS 4: centro-medial; mMS 5–6: left/right fronto-parietal. Increases in occipital alpha power in RCE relative to ROE correlated with greater mMS 1–2 time coverage (*τ*_*b*_s < 0.20, *p*s > .002), while the lateralization of deviance detection in MMN was associated with mMS 5–6 time coverage (*τ*_*b*_s < 0.16, *p*s > .012). No temporal correlation was found between EEG and MEG microstates (*p*s > .05), despite some overlap in brain sources and global explained variance between mMS 2–3 and EEG microstates B-C (*r*s > 0.60, *p*s < .002). Hence, the MEG signal can be decomposed into microstates, but mMS brain activity clustering captures phenomena different from EEG microstates. Source reconstruction and task-related modulations link mMS to large-scale networks and localized activities. Thus, mMSs offer insights into brain dynamics and task-specific processes, complementing EEG microstates in studying physiological and dysfunctional brain activity.

**Supplementary Information:**

The online version contains supplementary material available at 10.1007/s10548-024-01073-z.

## Introduction

In electroencephalography (EEG), the spontaneous organization of discrete spatial configurations over time has been studied by defining microstates. Microstates are transient patterns of scalp configurations of brain activity, lasting from 50 to 150 milliseconds (Michel and Koenig 2018). They have been hypothesized to derive from specific brain generators and functional networks, usually investigated by functional magnetic resonance imaging (fMRI). Neuroimaging revealed various resting-state networks associated with specific functions (e.g., somatomotor, visual, auditory, dorsal attention networks), including external stimulus processing or internal superior cognitive functions (Biswal et al. 1995; Deco et al. 2011; Seitzman et al. 2019). MEG provides superior source reconstruction compared to EEG and could enhance EEG microstate research with better-resolved spatial patterning (Hedrich et al. 2017). Despite extensive EEG research on microstates, the application of microstate analysis on MEG data is still sparse.

**Fig. 1:**
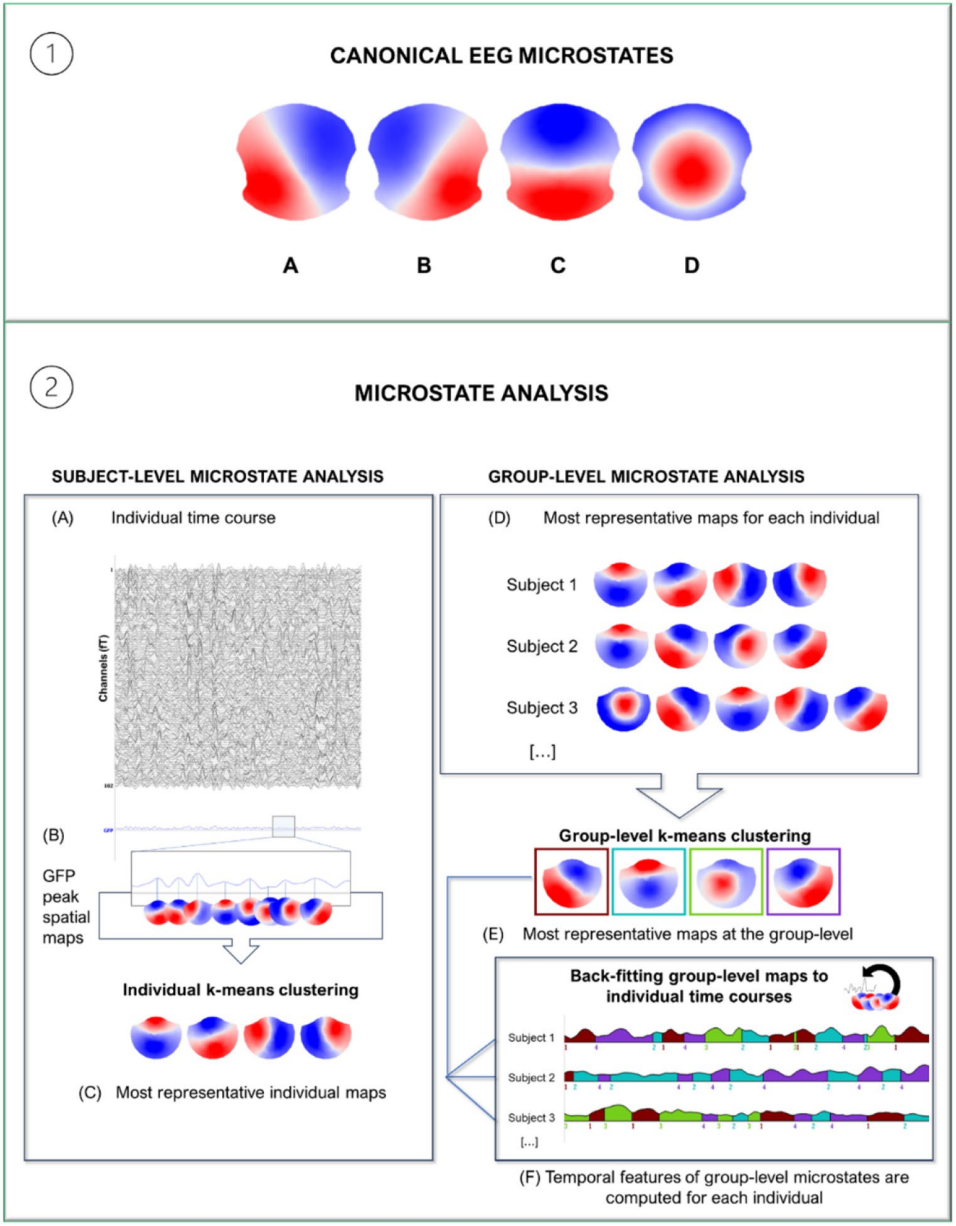
Panel 1. The four canonical EEG microstates, courtesy of Tomescu et al. (2018). Panel 2. Graphical representation of the standard procedure for extracting microstates from resting-state data, as applied in this study. The first step describes processes on single-subject data (i.e., multi-sensor acquisition). Spatial maps are computed at Global Field Power (GFP) peaks of the data (**A-B**), corresponding to the time frames with the highest signal-to-noise ratio. To identify the spatial maps that are the most representative of the single subject signal, these spatial maps feed the k-means clustering algorithm (**C**). The second step describes processes at the group level. The most representative maps of each individual (**D**) are the input of the group-level k-means clustering. The output of this process is a set of maps that represent all the data of the group (**E**). The group maps are then fitted to individual data, resulting in a sequence of microstates over time (**F**). From this individual sequence, microstate temporal features (e.g., time coverage) are computed for each subject and used for subsequent between-group or between-condition comparisons

Microstate configurations remain dominant for short periods (50–150 ms) and rapidly transition to another configuration, which in turn becomes dominant for a short duration. Thus, microstates are modeled such that only a single discrete global brain state occurs at any given time (Khanna et al. 2015). During the resting state, four dominant EEG microstates – conventionally labeled as A, B, C, and D - have been consistently described (Michel and Koenig 2018). These microstates exhibit left anterior/right posterior (microstate A), right anterior/left posterior (microstate B), anterior to posterior (microstate C) dipole orientations, and a central maximum (microstate D) (see Fig. 1, panel 1). Although many studies have used four clusters as a fixed number, primarily to facilitate comparability across different experiments, cross-validation methods can identify a dataset-specific optimal number of clusters. This approach typically leads to substantial improvement in the explained variance of the signal (Michel and Koenig 2018). Recently, a comprehensive systematic review conducted by Tarailis et al. (2024) updated the microstate classification with the introduction of three novel configurations alongside the four canonical microstates: a posterior variant of microstate C, as well as a left-lateralized and a right-lateralized microstate.

Attempts to understand the functional meaning of each microstate have included the definition of brain generators. In simultaneous EEG-fMRI signal analysis, Britz et al. (2010) showed that the four canonical microstates are associated with four functional brain networks: microstate A with the auditory network, microstate B with the visual network, microstate C with parts of the cognitive control and default mode networks, and microstate D with the dorsal attention network. Studies that reconstructed the brain sources of EEG microstates recorded with high-density EEG (Custo et al. 2017; Bréchet et al. 2019) partially replicated Britz et al.’s (2010) findings. Microstates A and B were associated with brain activity in regions involved in auditory and visual processing, respectively. Microstate D sources were localized in the right inferior parietal lobe and the right middle and superior frontal gyri, which are spatially congruent with the dorsal attention network. However, at variance with Britz et al. (2010), Microstate C generators were estimated in the precuneus and the posterior cingulate cortex in Custo et al. (2017) and bilaterally in the parietal cortex in Bréchet et al. (2019). Using source localization of EEG data, Pascual-Marqui et al. (2014) found substantial overlap between microstates. In addition to a shared hub in the posterior cingulate cortex, microstates A and B involved the left and right occipito-parietal cortex, and microstate C was attributed to sources in the anterior cingulate cortex. The heterogeneity of source-based reconstructions of EEG microstate activity may be related to the modest spatial resolution of the EEG signal and to small sample sizes, which may have limited the reproducibility of the findings; the time scale of higher-resolution fMRI signals, on the other hand, is too different from EEG to replicate the source reconstruction results.

An alternative approach to elucidate the functional role of EEG microstates has been to compare their spatial and temporal properties between resting with eyes open (ROE) and resting with eyes closed (RCE). Besides a reduction in total global explained variance in ROE compared to RCE (Seitzman et al. 2017; Zanesco et al. 2020), conflicting results have been obtained when examining differences between the temporal dynamics of specific microstates. Seitzman et al. (2017) reported a significant decrease in the duration of microstate D and an increase in the occurrence of microstate B in ROE compared to RCE. Instead, Zanesco et al. (2020) found an increase in the duration of microstate D but no change in the occurrence of microstate B. The association of microstate B with activity changes between resting-state conditions is further supported by the association of this microstate with alpha power (Croce et al. 2020), the frequency band that shows the most pronounced changes when transitioning from RCE to ROE (Adrian and Mathews 1934; Berger 1929).

Resting-state microstate configurations have also been investigated by examining possible differences between groups or variations of temporal parameters when fitted to the signal during the performance of a cognitive task. Microstate A has been mainly associated with phonological operations (Korn et al. 2021; Diaz et al. 2013, 2014) and, to a lesser extent, visual processing (Cui et al. 2021; Jabès et al. 2021; Zappasodi et al. 2019). However, increases in microstate A time coverage have been recently associated with arousal and alertness (Antonova et al. 2022; Ke et al. 2021; Jawinski et al. 2021). Microstate B appears robustly involved in visual processing and visuospatial attention (Seitzman et al. 2017). Microstate C functions may reflect mind-wandering and introspection (Tarailis et al. 2021). Microstate D appears to be related to executive function, working memory, and attention (Tarailis et al. 2024), although mixed interpretations have been reported in the literature (see Milz et al. 2016). These findings suggest that microstates A and B primarily involve sensory processing, whereas microstate C is associated with higher cognitive processes and microstate D with executive function and attention. However, results are heterogeneous, and differences in experimental paradigms limit the generalization of the findings.

Compared to EEG, MEG provides more accurate spatial and less noisy measurements of brain activity, especially with planar gradiometers (Garcés et al. 2017). These MEG features can potentially improve microstates’ spatial and temporal definition, allowing more precise identification of their corresponding cortical generators and functional roles. Only two methodological studies investigated MEG microstates, yet neither strictly employed the standard procedure for microstate analysis with Cartool (Brunet et al. 2011) commonly used in studies on EEG microstates.

Coquelet et al. (2022) investigated microstates and Hidden Markov Model states in simultaneous EEG-MEG recordings, applying a fixed number of microstates to hierarchical clustering. The four MEG microstates they obtained did not match EEG microstates regarding topographies and signal labeling. They also had poor temporal correspondence with Hidden Markov Model states. Additionally, MEG microstates were shorter (37 ms on average) than the commonly reported durations of EEG microstates (50–150 ms, Michel and Koenig 2018), thus describing quasi-instantaneous neurophysiological events in MEG. They concluded that microstates were not reproducible across imaging modalities. The observed inconsistency may be due to EEG and MEG capturing different yet complementary information from brain sources (Vrba and Robinson 2001), e.g., tangential sources in MEG and mainly radial sources in EEG (Corsi 2023). Moreover, while EEG microstates represent activity patterns with a reversal phase to the reference (Michel and Koenig 2018), MEG microstates resulting from combined planar gradiometers indicate patterns of positive activation independent from a reference.

Tait and Zhang (2022) overcome this potential incongruence between EEG and MEG signals at the scalp level by analyzing MEG region of interest (ROI) time courses to generate microstates in the source space. These microstates were characterized by specific patterns of phase-synchronized connectivity within four bilateral networks (frontal, fronto-temporal, visual, and orbital microstates) and three pairs of symmetrical unilateral networks (left and right parietal, left and right temporal pole, left and right sensorimotor microstates). At the functional level, auditory stimuli resulted in more frequent identification of the frontotemporal network 100 ms after stimulus onset, suggesting a sensitivity of cortical microstates to event-related brain responses (Tait and Zhang 2022).

The described MEG studies differed from previous EEG research in many methodological aspects since establishing a possible functional link between MEG and EEG microstates was not their objective. Identifying MEG microstates and understanding their relationships with EEG microstates might offer new means for microstate research in moving from the scalp to the brain sources, with important applications in characterizing abnormal microstate patterns in clinical populations.

The primary objective of this study was to determine the feasibility of extracting microstates at the sensor level from MEG signals, analyzing their neuronal substrates and functional characteristics. Additionally, we investigated potential relationships between MEG and EEG microstates by comparing signal labeling, brain sources, and global explained variance (GEV) in simultaneously recorded EEG and MEG data. To this end, we carried out two experiments applying microstate analyses to two different MEG datasets: one on multi-task MEG data collected by the Group of Psychiatric Neuroscience of the University of Bari (Italy) and one on resting-state EEG/MEG data collected by the Clinical Neurophysiology Research Laboratory of the University of Pittsburgh (PA, USA). The first experiment aimed to identify resting-state MEG microstates, following the methodology used to compute EEG microstates as closely as possible, and investigate their underlying brain sources and functional significance. To this end, we segmented the MEG signal by fitting resting-state MEG microstates to the MEG signal recorded during two resting-state conditions (ROE and RCE) and an auditory Mismatch Negativity task (MMN). For labeled time frames, we performed (1) source reconstruction, (2) computed the average activity in parcels of the brain surface corresponding to different functional networks, and (3) tested possible changes in MEG microstate time coverage during different resting-state and task conditions, exploring possible associations of MEG microstates with alpha power during RCE and stimulus processing in MMN. The second experiment aimed to replicate resting-state MEG microstates in an external sample. Moreover, we explored the relationship between EEG and MEG microstates by assessing the overlap of microstate sequences, corresponding reconstructed brain sources, and microstate features.

## Experiment 1

### Methods

#### Participants

The participants included in this study were a subset of individuals from the control groups reported by Valt et al. (2023) in their investigation of MMN in psychosis. Of the 135 control participants, only 113 individuals had usable resting-state recordings. Therefore, the sample size for the current study consisted of 113 healthy participants with a mean age of 26.5 years (SD 6.8), of whom 58.3% were female. Participants were required to be unmedicated and have no history of neurological or psychiatric disorders. Additionally, a trained psychologist conducted a Structured Clinical Interview for DSM-5 (First et al. 2017) to confirm the absence of any relevant psychiatric conditions.

The Ethics Committee of the University Hospital of Bari approved the MEG protocol used in this study, and all participants gave informed consent for the MEG data collection.

#### Procedure

The experimental protocol included two 5-minute resting-state conditions and two tasks. The two resting-state conditions were one with eyes open (i.e., ROE) and one with eyes closed (i.e., RCE). The tasks were an auditory mismatch negativity (i.e., MMN) task with duration-deviant stimuli (Näätänen et al. 2007) and a visual task designed to evoke a 10 Hz visual steady-state potential. The visual task was not analyzed as part of this study.

During the resting-state conditions, participants were instructed to remain still throughout the recording session. They were explicitly advised not to engage in any particular thoughts, to avoid nodding off, and to avoid falling asleep. During the ROE, participants were required to keep their eyes open and focus on a fixation cross displayed on a screen, while during the RCE, participants were instructed to keep their eyes closed.

In the MMN task, participants were asked to watch a silent Tom & Jerry cartoon while a sequence of 667 auditory stimuli was presented to both ears through earphones. All the auditory stimuli had a frequency of 1000 Hz and an intensity of 80 dB, while the duration was 50 ms for 612 stimuli (standard) and 100 ms for 55 stimuli (deviant). Deviant and standard stimuli were pseudorandomly intermixed. The interstimulus interval between the tones was 500 ms.

#### MEG Data Recording and Processing

MEG signals were acquired using a 306-channel Elekta Neuromag^®^ TRIUX whole-head system (Elekta Neuromag Oy, Helsinki, Finland) with a recording sampling rate of 1000 Hz. The MEG signal was processed offline with Elekta MaxFilter™ software from Elekta Neuromag Oy (Helsinki, Finland) to remove external noise with temporal Signal-Space Separation (tSSS), as described by Taulu and Simola (2006), and to correct for head motion using data from five Head Position Identifier coils. After these preliminary preprocessing steps, the MEG signal was further processed using Fieldtrip (Oostenveld et al. 2011) to prepare the ROE data for MEG microstate identification or with Brainstorm (Tadel et al. 2011) to prepare the ROE, RCE, and MMN data for MEG microstate fitting for source reconstruction, network activity analysis, alpha power calculation, and MMN peak amplitude extraction. The processed data, converted to a BrainVision format, were then imported into the Cartool software (64-bit release version 4.10. Revision 7573; Brunet et al. 2011) for all the microstate analyses.

Data were down-sampled to 250 Hz, and frequencies associated with the European power grid (notch filter: 50 Hz), low frequencies (high-pass filter: 1 Hz) and high frequencies (low-pass filter: 30 Hz) were removed. Artifacts related to eye movements and heartbeat were identified and corrected using Independent Component Analysis (ICA) trained on all the sensors. To ensure optimal signal quality for subsequent microstate identification, the ROE data were segmented into 2-second time windows. Subsequently, epochs containing potential artifacts were identified and rejected using Fieldtrip’s automatic artifact rejection function (*ft_artifact_zvalue*). The resulting cleaned epochs (corresponding to 242.3 ± 29.6 s of clean recording on average) were concatenated. For each sensor, gradiometer pairs were combined to obtain the planar gradient magnitude over both directions by computing the square root of the sum of the squares for each gradiometer pair – as reported by Coquelet et al. (2022). Magnetometers were not considered to prevent potential problems arising from differences in the scale of the two sensor types.

#### MEG Microstate Identification

A spatial filter was first applied to smooth topographies and remove outliers (Michel and Brunet 2019). The spatial filter determines and interpolates the maximum and minimum values from the six nearest neighboring sensors for each location. Maps were then extracted at peaks in the Global Field Power (GFP) of the data, which are time frames with the highest signal-to-noise ratio. K-means clustering was then applied to the resulting collection of maps.

Cartool’s default parameters for resting-state MEG analysis were used for k-means clustering, i.e., only positive values, no average reference, and the polarity of the data not being relevant. K-means clustering (see Fig. 1, Panel 2) was applied to individual ROE data to estimate the optimal set of maps for each subject (individual level) and then across all participants to estimate the optimal set of maps for the entire dataset (group-level). For both levels of clustering, the optimal number of clusters was determined automatically via a meta-criterion validation (Michel and Koenig 2018). This cross-validation method determines the optimal k-number as the median output of different validated criteria (i.e., Gamma, Silhouettes, Davies and Bouldin, Point-Biserial, Dunn, Kraznowski-Lai Index, and the median value of these criteria). The maps obtained at the group level were the MEG microstate used for all subsequent analyses.

#### MEG Microstate Fitting

The MEG microstates identified from the ROE recordings were fitted to each individual recording (ROE, RCE, and MMN). This procedure assigned the microstate with the highest spatial correlation to each time frame (i.e., competitive fitting) only if the correlation between the data topography and the microstate topography was above the minimum threshold of 0.50. Consequently, some parts of the data may remain unlabeled. Temporal smoothing was applied (window half size = 3 time frames, Besag factor = 10). After the fitting procedure for each microstate, the proportional time coverage was calculated as the sum of the time points labeled by each microstate divided by the total number of time points in the data.

#### Source Reconstruction of MEG Microstates

The individual inverse source model was constructed as overlapping spheres with 15,002 vertices, with a noise covariance matrix derived from a 2-min empty room recording, which was acquired and processed with the same settings used while the participants were performing the tasks. After linear scaling, the ROE sensor-based signals were converted to source-based signals with standard Low-Resolution Brain Electromagnetic Tomography (sLORETA, Pascual-Marqui 2002). The brain cortex model included constrained vertices extracted from the ICBM152 MRI template (Mazziotta et al. 2001). The source-based data corresponding to periods labeled by a given MEG microstate were averaged with each time frame weighted by the correlation between data topography and microstate topography. The average source activity of the whole signal was subtracted from the average source activity of each MEG microstate to isolate the specific activation associated with each MEG microstate.

In addition, for each MEG microstate, we averaged the source-estimated signal over labeled time frames in each of the 34 parcels (17 parcels for each hemisphere) of Schaefer’s brain atlas (Schaefer et al. 2018). This brain atlas parcellates the cerebral cortex into brain networks (e.g., Default Mode, Saliency, etc.) based on functional connectivity data from neuroimaging studies.

#### Alpha Power Computation

To assess the relationship between MEG microstate modulations between ROE and RCE and occipital alpha power enhancement when participants kept their eyes closed compared to when they kept their eyes open, the frequency power of activity at the right and left occipital sensors in ROE and RCE was calculated using Fast Fourier Transformation. The resulting frequency power spectra were then averaged over the selected sensors. To quantify the change in occipital alpha activity during RCE compared to ROE, the average frequency power of RCE was subtracted from the average frequency power of ROE. The amplitude of the most prominent negative peak within the 8 to 12 Hz frequency range was used as an index of the increase in occipital alpha activity during RCE.

#### MMN Peak Amplitude Computation

To investigate potential variations in MEG microstate detection rates during the MMN time window, the number of segments labeled by a specific MEG microstate at a given time point from 150 ms to 300 ms after stimulus onset was calculated for 55 standard and 55 deviant auditory stimuli. Thus, the maximum detection rate in a time point could be 55, meaning that a specific MEG microstate was identified in all the segments at that specific time point.

We then assessed whether MEG microstate labeling was sensitive to detecting MMN amplitude differences between the left and right auditory regions. For the MMN peak amplitude computation, the MMN source-based signal was divided into epochs starting 200 ms before stimulus onset and lasting 700 ms. The potentials of deviant stimuli and standard stimuli preceding a deviant stimulus were then normalized to z-scores based on the 200 ms pre-stimulus interval, converted to absolute values, and spatially smoothed before averaging. The MMN peak amplitude was determined as the most prominent negative peak between 150 and 300 ms in the subtraction of deviant from standard activity reconstructed to vertices of parcels of the Destrieux atlas (Destrieux et al. 2010). These parcels correspond to the left and right primary and secondary auditory regions, i.e., the superior temporal gyrus/*planum temporale* parcel and the temporal transverse sulcus parcel.

#### Statistical Analysis on MEG Microstates

For the network analysis, to determine whether there was an increase or decrease of microstate-related mean activity in specific network parcels of the brain surface, we computed a null distribution of activities in the brain parcels extracted from 1000 permutations of random assignments of microstates to the data, preserving the number and duration of the identified MEG microstates. Afterward, the association of a MEG microstate with a specific network was determined by comparing the normalized mean signal amplitude in a specific parcel with the corresponding normalized null distribution. Observations significantly different from the mean of the null distribution were determined according to the critical |Z-score| of 3.6. This Z-score corresponds to a Bonferroni corrected p-value of 0.0002 (α/number of comparisons = 0.05/(34*6)).

For the analysis of MEG microstate temporal features, we computed the time coverage as the percentage of signal labeled by a specific MEG microstate in ROE, RCE, and MMN. Since time coverage does not fulfill the ANOVA requirement of independence between observations, contrasts between ROE and RCE and between ROE and MMN were performed with microstate-specific t-tests, with the α of 0.05 adjusted with Bonferroni’s correction to account for multiple comparisons (α/number of comparisons: 0.05/6 = 0.008).

In RCE, besides the mMS-specific tests contrasting ROE and RCE, we correlated with Kendall’s tau the differences in individual microstate time coverage between ROE and RCE with the differences in mean alpha power across occipital sensors between ROE and RCE to evaluate the sensitivity of MEG microstates to changes in occipital alpha activity.

In MMN, besides the contrasts of MEG microstate time coverages between ROE and MMN, additional t-tests investigated stimulus-related differences by considering variations in the average frequency of MEG microstate detections during the 150–300 ms time window after the onset of standard or deviant stimuli. Furthermore, we investigated the MEG microstate’s sensitivity to the MMN laterality. To this end, we correlated with Kendall’s tau the difference in MEG microstate detection rates between standard and deviant stimuli, termed MEG microstate mismatch negativity (mMS-MMN), with the discrepancy in MMN peak amplitude between the right and left auditory regions (MMN lateralization score). A positive correlation between mMS-MMN and MMN lateralization scores would indicate a MEG microstate sensitivity to increase MMN amplitudes over the right auditory region. In contrast, a negative correlation would indicate a microstate sensitivity to increase MMN amplitudes over the left auditory region.

## Results

### MEG Microstate Identification

**Fig. 2:**
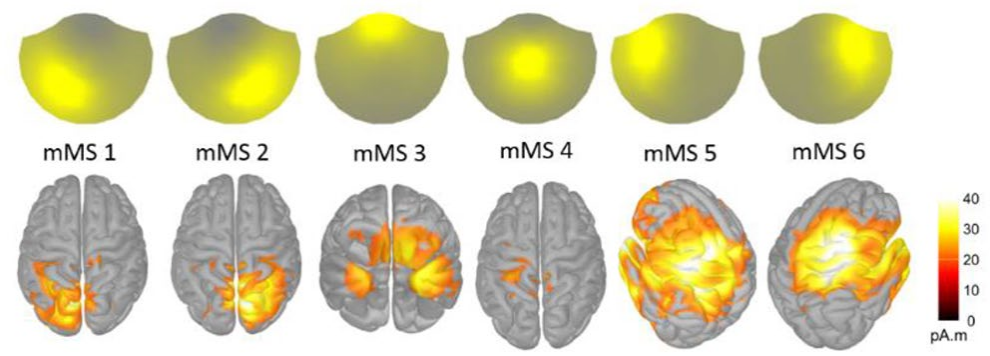
Topographies of the six MEG microstates (mMS) and corresponding source-based activity of the MEG signal labeled by each mMS. The six MEG microstates were named from mMS 1 to mMS 6

K-means clustering on ROE activity identified six distinct MEG microstates as the best fit to the data based on the meta-criterion (Fig. 2). Among these MEG microstates (magnetic microstates, mMS), four showed topographies similar to canonical EEG microstates (mMS 1–4). In contrast, two (mMS 5–6) showed fronto-lateral right and left patterns, respectively. Notably, similar topographical distributions between EEG and MEG microstates do not imply signal source similarity, as the native EEG and MEG signals are shifted of 90-degrees. On average, the percentage of ROE time frames labelled by the six mMSs was 70% (*SD* = 10%, range 50-96%), with an average time coverage for each mMS of approximately 12% and an average duration of 87.4 ± 7.3 ms.

### MEG Microstate Source Reconstruction

Descriptively, source-based analyses of the six MEG microstates (Fig. 2) showed left and right occipito-parietal activity for mMS 1 and mMS 2, respectively, a ventral fronto-temporal activity for mMS 3, a centro-medial activity for mMS 4, and left and right fronto-parietal activity along the central sulcus, which extended to the superior temporal gyrus, for mMS 5 and mMS 6, respectively.

Mean activity in time frames labeled by an mMS was compared with the mean activity of the normalized null distribution of randomly selected time frames to determine microstate-related activity changes in parcels of the brain surface that reflect distinct functional brain networks. Table 1 reports the networks that showed a significant difference from random activity. Interestingly, for each mMS, there were statistically significant increases as well as decreases in specific brain parcels compared to random (reported in Table 1 as + and -, respectively).

**Table 1:**
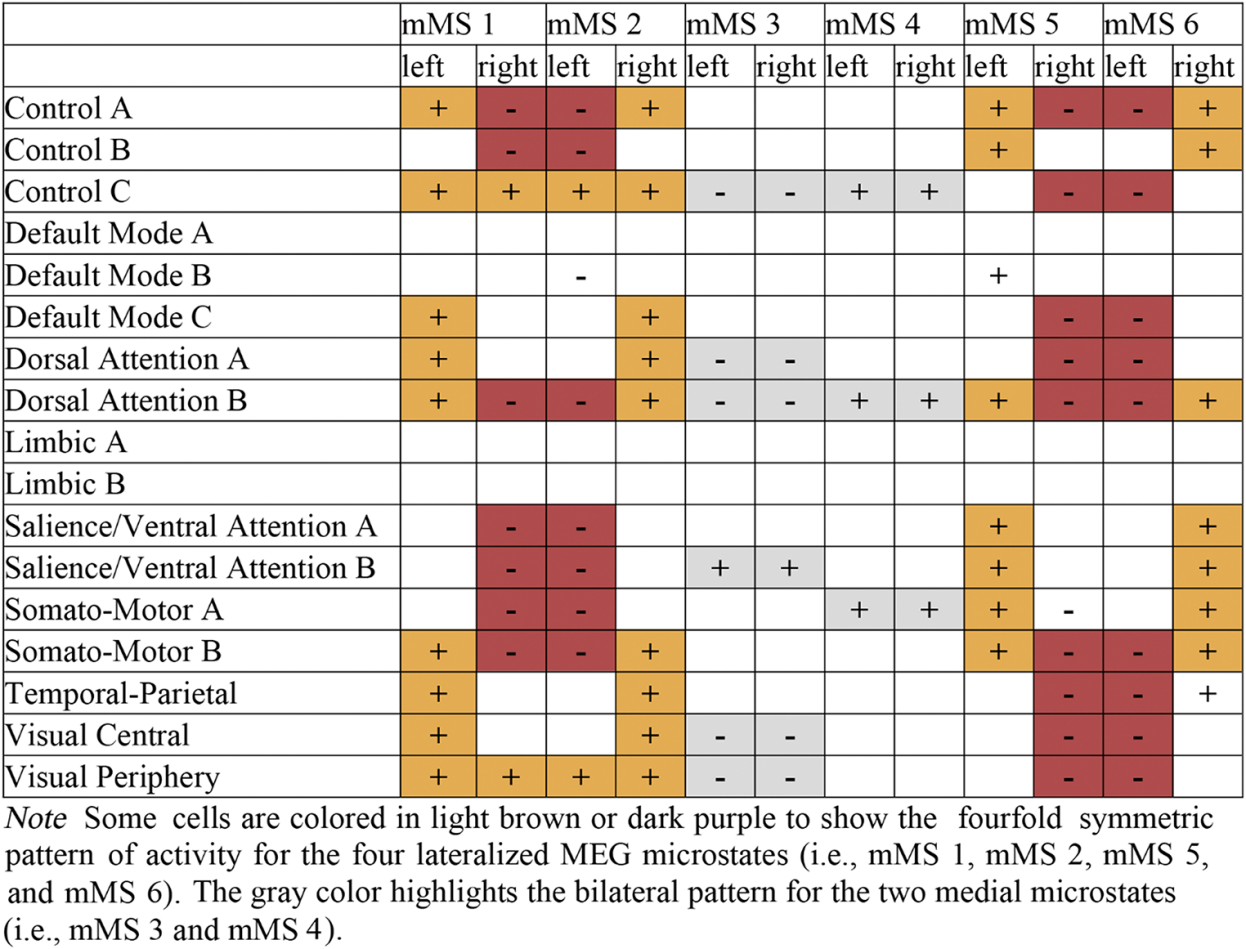
Networks showing greater (+) or less (-) than random activity in parcels of the brain surfaces for source-reconstructed data labeled by an MEG microstate

This analysis indicated that, overall, mMS 2 and mMS 6 showed greater activity in networks of the right hemisphere and less activity in networks of the left hemisphere. Conversely, mMS 1 and mMS 5 mainly presented increased activity in networks of the left hemisphere and decreased activity in networks of the right hemisphere. The pattern of activity was bilateral for mMS 3 and mMS 4.

Interestingly, most networks that showed less activity in mMS 5 and mMS 6 presented greater activity in mMS 1 and mMS 2, respectively. This fourfold symmetry (see colors in Table 1) applied to mMS 1, mMS 2, mMS 5, and mMS 6 in the three Control networks, the Default Mode C network, the two Dorsal Attention networks, the two Salience/Ventral Attention networks, the two Somato-Motor networks, the Temporal-Parietal network, and the two Visual networks. In general, mMS 5 and mMS 6 captured mostly increases in ipsilateral fronto-central activity from Control A, Control B, Dorsal Attentional B, Saliency/Ventral Attention, and Somato-Motor networks, whereas mMS 1 and mMS 2 captured mostly increments of ipsilateral posterior and medial occipito-parietal activity from Control A, Default Mode C, Dorsal Attention, Somato-Motor B, Temporal-Parietal, and Visual networks. For mMS 1 and mMS 2, there were also control-lateral activations in Control C and Visual Periphery. Networks that showed changes of activity not congruent with this fourfold pattern were the Default Mode B network, which showed a deactivation for mMS 2 and an activation for mMS 5, and the Somato-Motor A network, which showed a deactivation for mMS 5.

In contrast to the strong lateralization of mMS 1, mMS 2, mMS 5, and mMS 6, bilateral activity patterns characterized mMS 3 and mMS 4. In the data labeled by mMS 3, the bilateral Salience/Ventral B networks were the only ones to show greater activity, whereas less activity was recorded in the bilateral Control C network, the two Dorsal Attention networks, and the two Visual networks. The signal labeled by mMS 4 showed greater than random activity only in the bilateral Control C networks, the Dorsal Attention B networks, and the Somato-Motor A networks.

### MEG Microstates in RCE

**Fig. 3:**
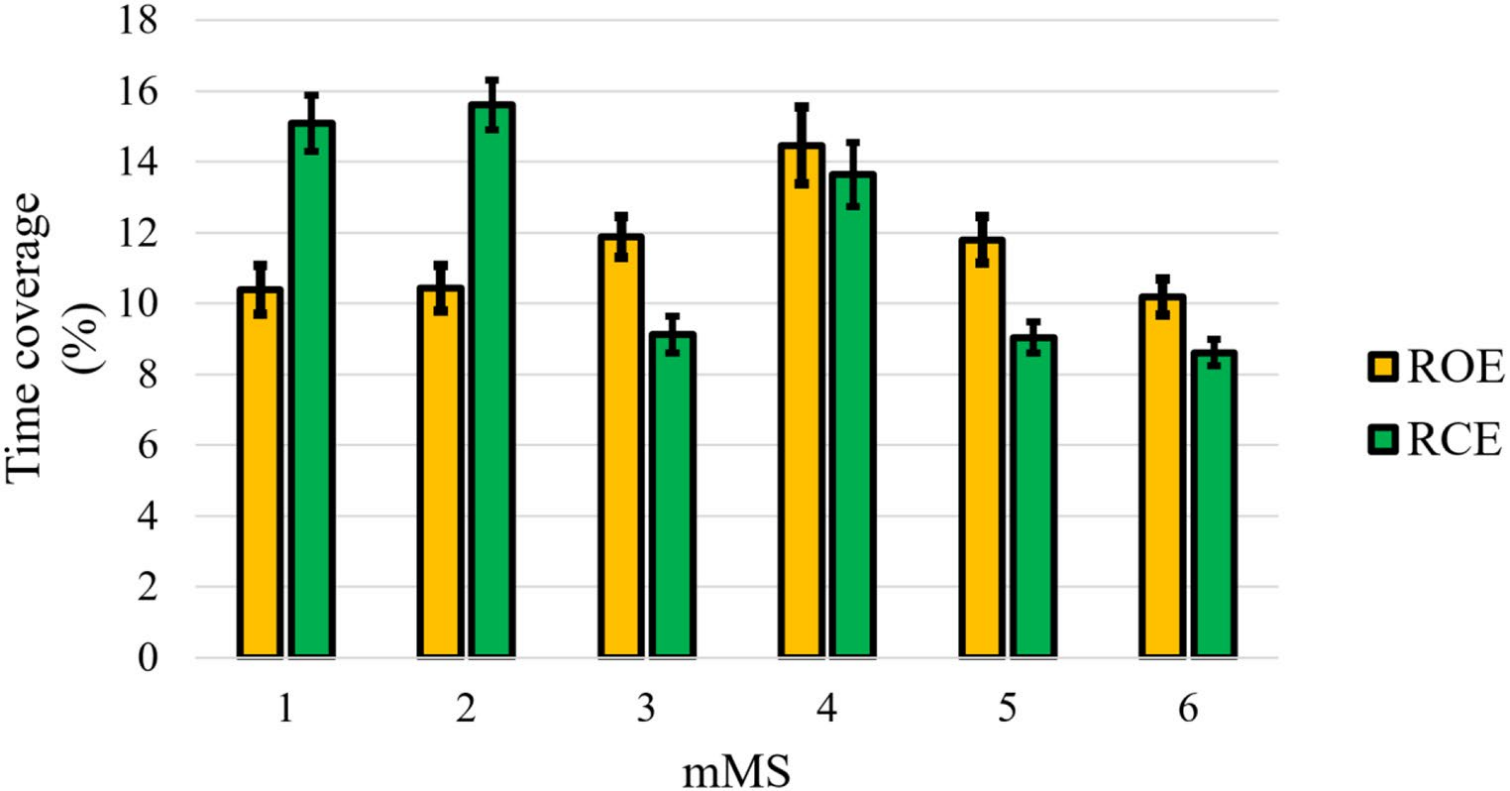
Mean time coverage of the six mMSs in ROE and RCE. Error bars represent standard errors of the mean

Microstate-specific contrasts between ROE and RCE showed (see Fig. 3) that the time coverage of mMS 1 and mMS 2 was significantly larger in RCE than ROE, *t*s(112) > 6.31, *p*s < .001, *d*s > 0.59, but it was significantly smaller for mMS 3, mMS 5 and mMS 6, *t*s(112) > 3.15, *p*s < .001, *d*s > 0.30. The time coverage of mMS 4 did not differ significantly between the two resting-state conditions, *t*(112) = 0.85, *p* = .396.

**Fig. 4:**
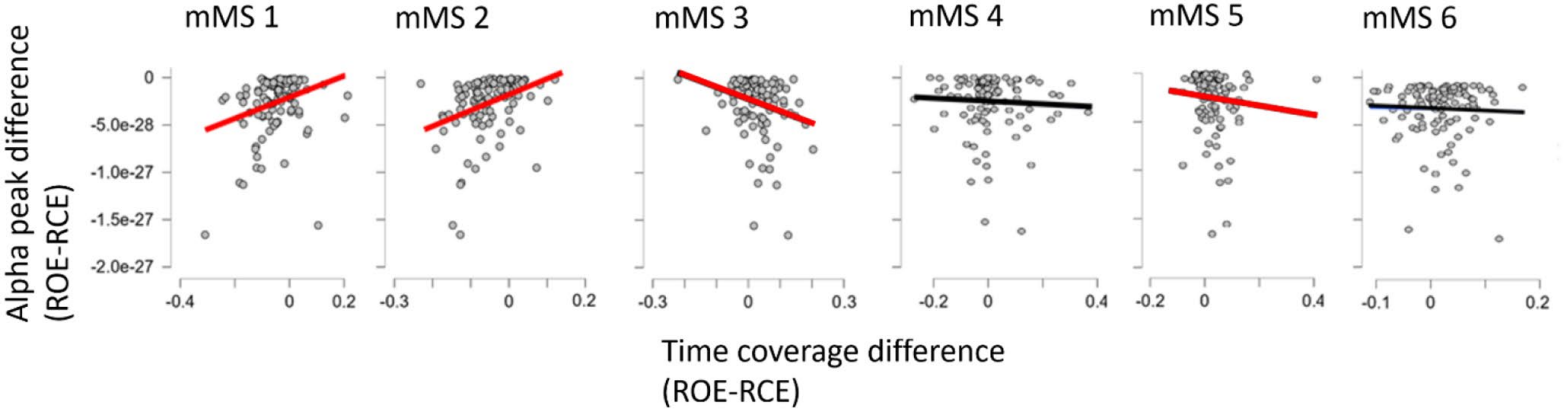
Scatter plots of changes in alpha power between ROE and RCE and changes in mMS time coverage between ROE and RCE. Red regression lines highlight significant correlations between the two factors

The time coverage differences between RCE and ROE of mMS 1, mMS 2, mMS 3 and mMS5 were significantly correlated with alpha power differences between RCE and ROE over the occipital sensors (see Fig. 4). Positive correlations were found for mMS 1, *τ*_*b*_ = 0.201, *p* = .002, and mMS 2, *τ*_*b*_ = 0.308, *p* < .001, indicating that increases of alpha power in RCE resulted in prolonged time coverage of these two posterior MEG microstates in RCE compared to ROE. In contrast, a negative correlation was found for mMS 3, *τ*_*b*_ = − 0.256, *p* = .006, and mMS 5, *τ*_*b*_ = − 0.162, *p* = .004, suggesting that occipital alpha activity during RCE decreased the time coverage of these anterior microstates.

### MEG Microstates in MMN

**Fig. 5:**
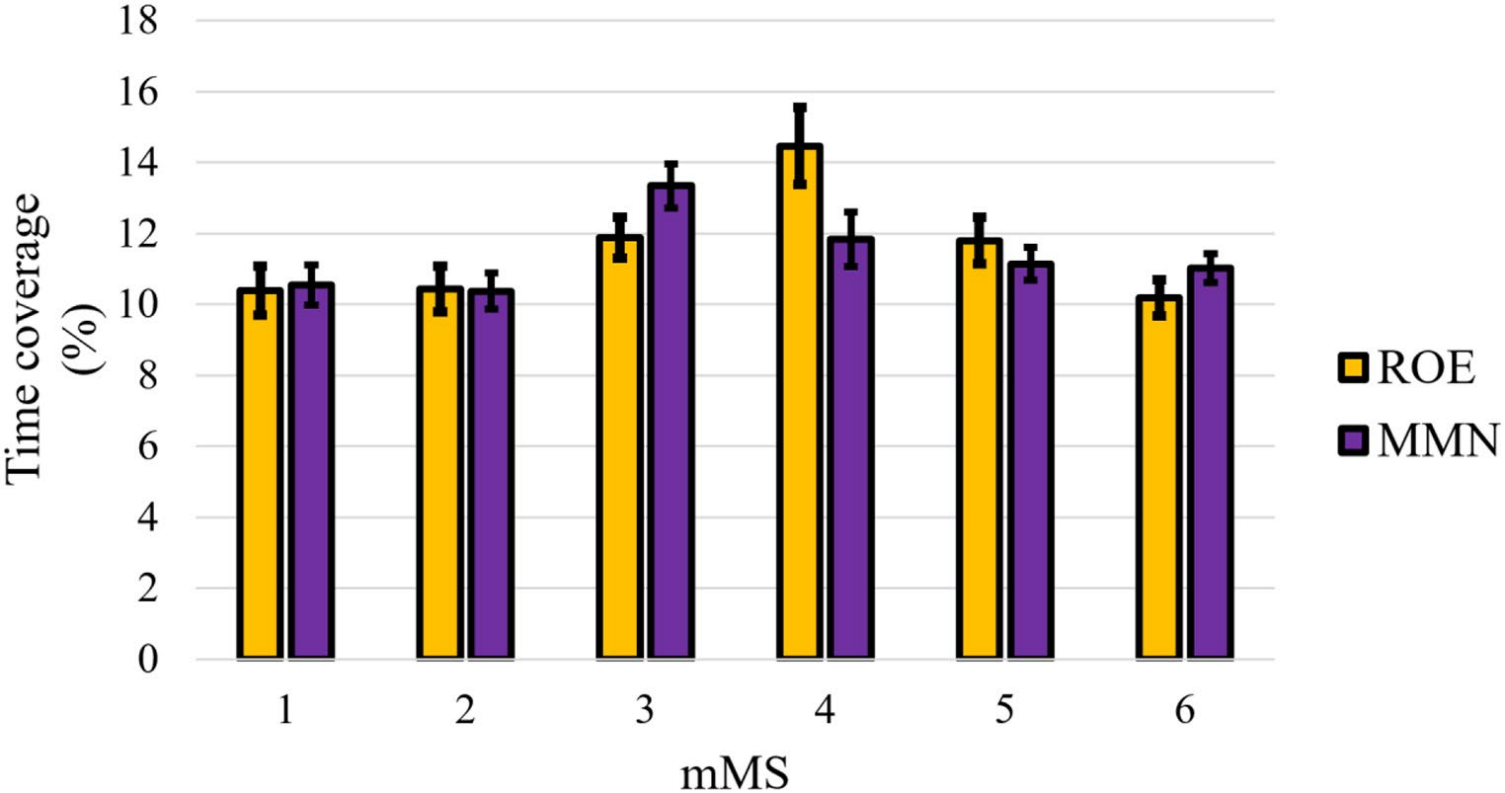
Mean time coverage of the six mMSs in ROE and MMN. Error bars represent standard errors of the mean

Microstate-specific contrasts between ROE and MMN (see Fig. 5) indicated that mMS 3 had larger time coverage during MMN compared to ROE, *t*(112) = 2.91, *p* = .031, *d* = 0.21, but mMS 4 had smaller time coverage during MMN compared to ROE, *t*(112) = 2.42, *p* = .017, *d* = 0.23 (all other contrasts, *t*s(112) < 1.53, *p*s > .128). However, neither of these two results survived correction for multiple comparisons.

**Fig. 6:**
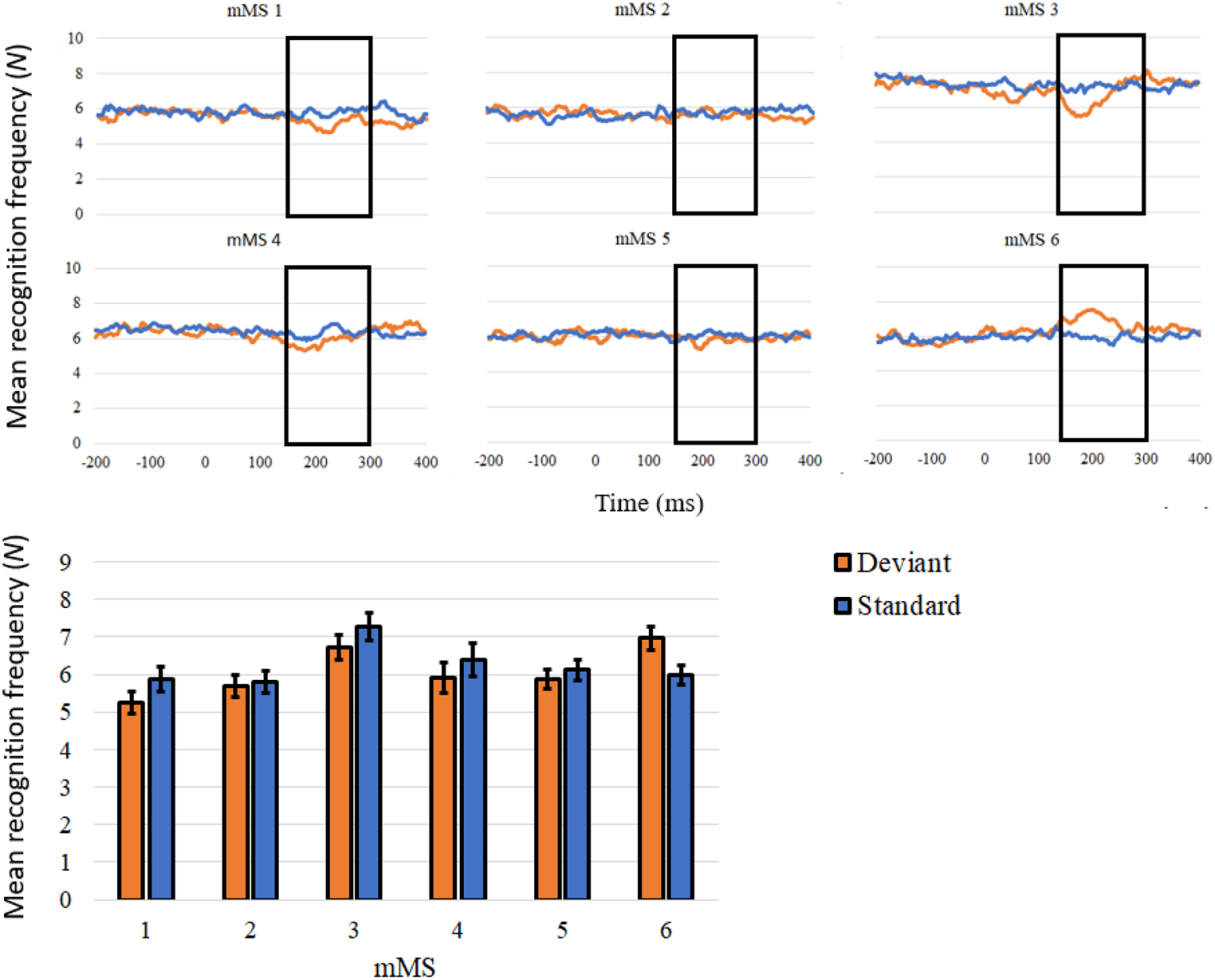
The line plots illustrate the frequency of recognition of a specific MEG microstate in each time-point of the extracted stimulus-locked segments, with a maximum of 55 epochs. The black rectangles frame the time window of deviance detection (150–300ms). The bar plot depicts the mean count of MEG microstate identification in the 150-300ms time window. Error bars represent the standard errors of the mean

In the analysis of MEG microstate recognition frequency in the signal evoked by standard and deviant stimuli, MEG microstates showed a sensitivity to the type of auditory stimulus in the time-window 150–300 ms after stimulus onset (Fig. 6). Compared to standard stimuli, deviant stimuli presented a higher detection rate of mMS 6, *t*(112) = 4.92, *p* < .001, *d* = 462, and a lower detection rate of mMS 1, mMS 3, and mMS 4, *t*s(112) > 3.24, *p*s < .002, *d*s > 0.31.

**Fig. 7:**
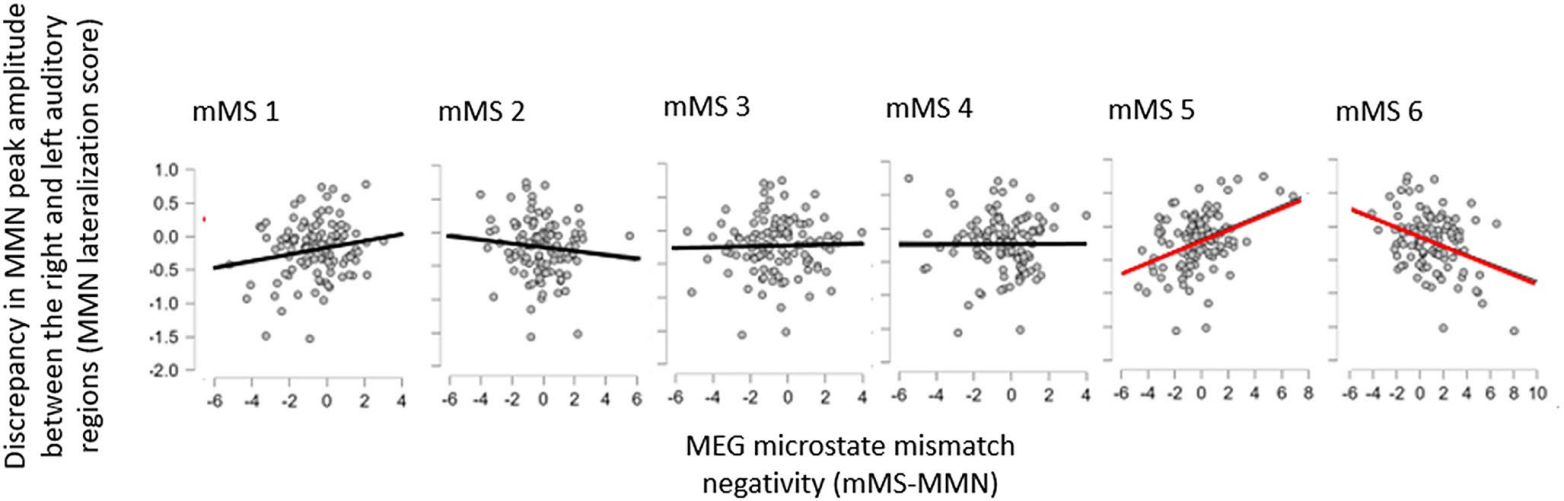
Scatter plots of MMN amplitude differences between right and left auditory regions and changes in mMS detection frequencies between standard and deviant stimuli. On the y-axis, negative and positive values indicate MMN lateralization to the right and the left, respectively. On the x-axis, positive values indicate more recognitions of a microstate for deviant than standard stimuli. Red regression lines highlight significant correlations between the two factors

Interestingly, there were also significant correlations between MMN-lateralization and changes in mMS detection rates for standard and deviant stimuli (Fig. 7). Specifically, mMS 6 was detected more frequently in deviant than in standard trials the more the MMN amplitude was lateralized to the right, *τ*_*b*_ = − 0.16, *p* = .012; conversely, when the MMN was lateralized to the left, there was an increase in the detection rates of mMS 5, *τ*_*b*_ = 0.22, *p* < .001.

## Experiment 2

### Methods

#### Participants

The sample for this experiment consisted of 21 healthy participants with complete EEG-MEG data. Participants had a mean age of 25.3 years (*SD* = 4.6), and 38.1% were female. They were required to be unmedicated and have no history of neurological or psychiatric disorders.

The Institutional Review Board of the University of Pittsburgh approved this study. All participants gave written informed consent and received compensation for participating in the MEG experiment.

#### Procedure

Participants performed a battery of cognitive tasks during simultaneous EEG-MEG acquisition, along with 5 min and 30 s resting state with open eyes (ROE).

#### EEG-MEG Data Recording and Processing

Data were acquired at the University of Pittsburgh Medical Center from a 306-channel Elekta Neuromag^®^ TRIUX whole-head system (Elekta Neuromag Oy, Helsinki, Finland) and 64 low-impedance EEG electrodes mounted on an elastic cap (BrainCap MEG, Brain Vision, LLC, Morrisville, NC, USA) according to the 10–10 EEG system.

Data processing followed the same methodology as previously outlined in Experiment 1, treating EEG and MEG data separately. Artifact detection was performed on both EEG and MEG signals, and segments with artifacts were rejected from both modalities. The artifact-free signal was, on average, 301.0 ± 29.6 s.

#### EEG-MEG Microstate Identification and Microstate Fitting

Microstate analysis followed the pipeline detailed in Experiment 1. EEG microstate extraction followed Cartool’s EEG presets for k-means (i.e., signed values, average reference). Meta-criterion validation (Michel and Koenig 2018) was independently applied to EEG and MEG data to identify the optimal number of clusters.

#### Source Reconstruction of MEG and EEG Microstates

The individual inverse source model with 15,002 vertices was constructed as overlapping spheres for the MEG signal and a 3-shell sphere for the EEG signal. The noise covariance matrix was directly derived from the ROE data. Source conversion was performed separately for EEG and MEG data, with the transformation to absolute values applied only to reconstructed MEG sources. Since the percentage of signal labeled by microstates can substantially vary between the two modalities, signal subtraction was avoided to ensure visual comparability of brain sources. Moreover, given the rapidly decreasing sensitivity of MEG to progressively deeper brain sources (Singh 2014), we opted to reconstruct both EEG and MEG signals on the cortical surface areas.

#### Statistical Analysis

The temporal correspondence of EEG and MEG microstates was determined by computing the number of occurrences of EEG microstates when a specific MEG microstate was identified. To this end, we first identified the time frames labeled by a specific microstate in the MEG segmentation and then analyzed the microstate labeling in the corresponding time frames in the EEG segmentation. These values were then contrasted against the null distribution obtained by 10,000 permutations of random assignments of MEG microstates to the data, preserving the number and duration of the identified microstates. Individual Z-scores were averaged across participants. Mean Z-scores larger than 3 indicated a significant association between a MEG microstate and a specific EEG microstate.

The link between EEG and MEG microstates was further explored by correlating their global explained variance (GEV), which represents the overall proportion of variance in the signal accounted for by the identified microstates. Only positive correlations that survived correction for multiple comparisons were considered to establish a link between EEG and MEG microstates.

### Results

**Fig. 8:**
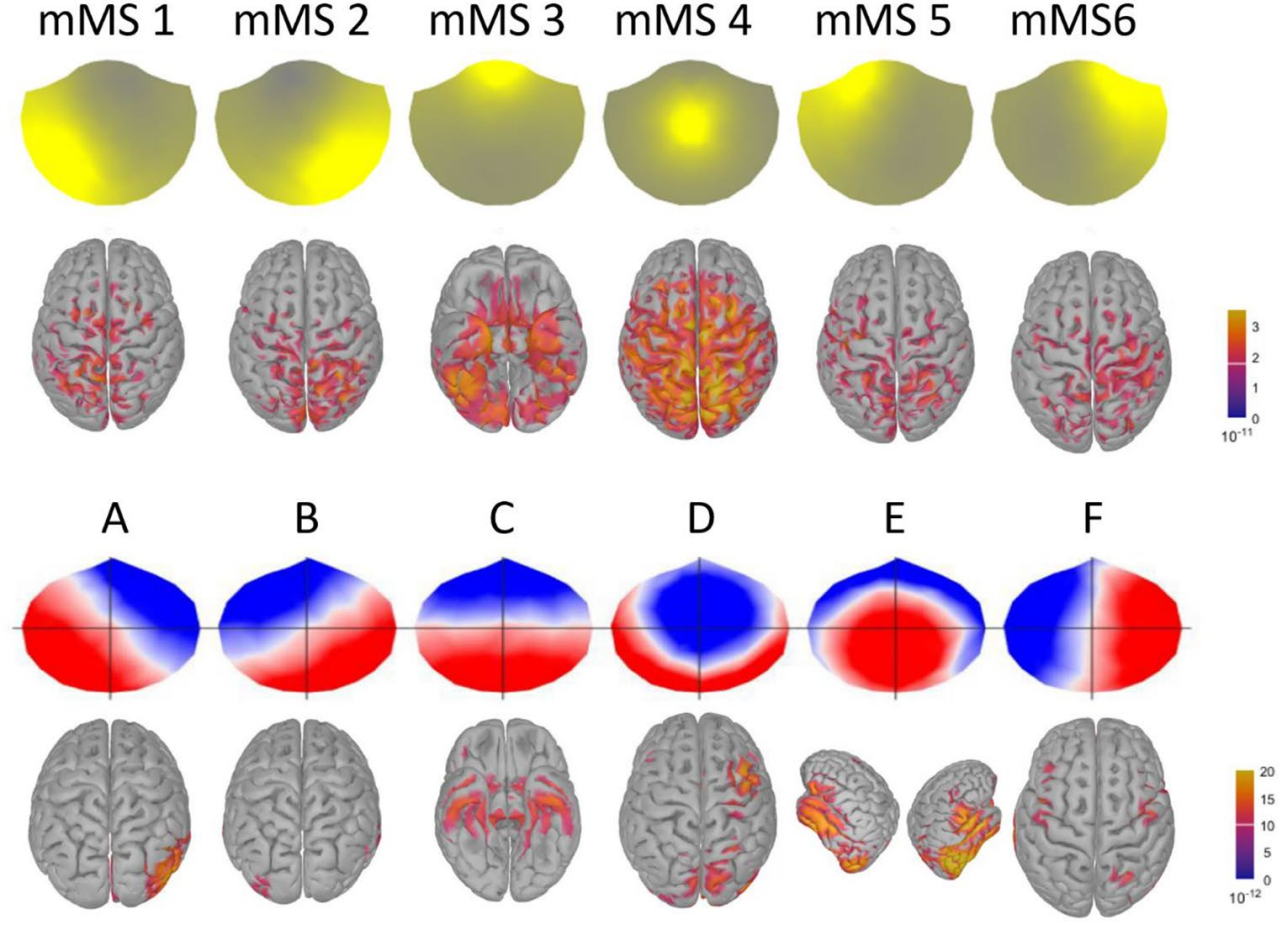
Topographies of the six MEG and EEG microstates, along with the corresponding source-based activity of the signal labeled by each microstate. The six EEG microstates were named based on visual similarity to canonical EEG microstate topographies (Tarailis et al. 2024)

K-means clustering on ROE activity determined that six MEG microstates and six EEG microstates were the best fit to the MEG and EEG data based on the meta-criterion (Fig. 8). Descriptively, the identified MEG microstate topographies resembled those found in Experiment 1. The six EEG microstates resembled the topographies of six of the seven topographies of the extended EEG microstate set, as reviewed by Tarailis et al. (2024).

On average, the percentage of ROE time frames labeled by the six mMSs was 56% (*SD* = 2%, range 51-61%), with an average time coverage of 11% for mMS 1, 11% for mMS 2, 9% for mMS 3, 6% for mMS 4, 10% for mMS 5, and 10% for mMS 6. EEG microstates labeled on average 99% (*SD* = 0.3%, range 98-99%) of the ROE signal, with an average time coverage of 15% for EEG microstate A, 17% for EEG microstate B, 28% for EEG microstate C, 13% for EEG microstate D and the other two EEG microstates.

The source-reconstructed activity of MEG microstates was congruent with the brain activity reconstructed in Experiment 1 (see Figs. 2 and 8): left and right lateralized occipito-parietal activity for mMS 1 and mMS 2, ventral fronto-temporal activity for mMS 3, centro-parietal activity for mMS 4, left and right fronto-parietal activity for mMS 5 and mMS 6, respectively. For EEG microstates, A and B were associated with right and left occipital sources, respectively, C with bilateral ventral fronto-temporal sources, D with right frontal and occipital sources, E with bilateral temporal sources, and F with bilateral frontal and right parietal sources.

**Fig. 9:**
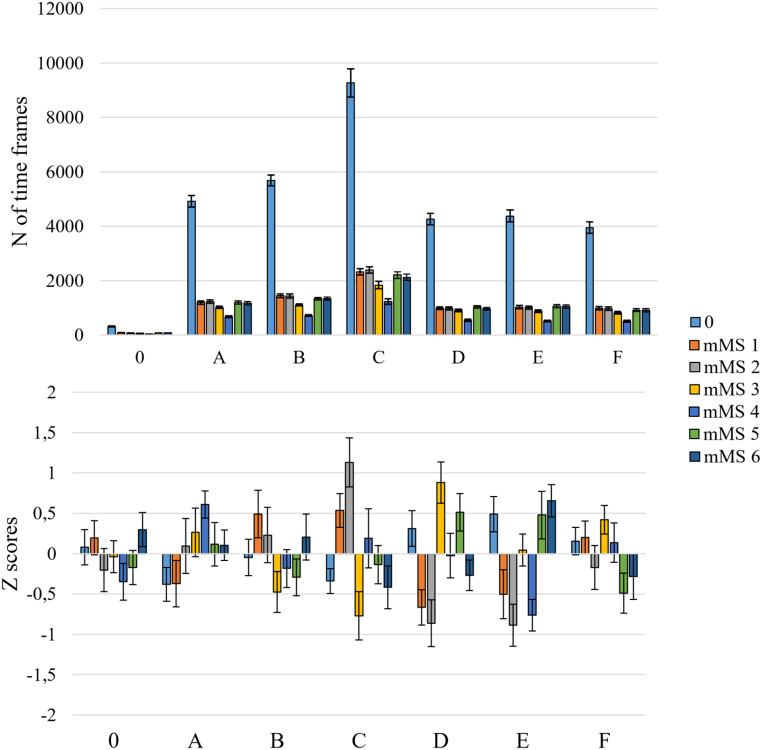
Upper panel: the mean count of time frames assigned to EEG microstates, partitioned according to the labeling of the MEG signal. Lower panel: z-scores of the count of time frames for the different combinations of EEG and MEG microstate occurrences. The unlabeled signal is reported as 0. Error bars represent the standard errors of the mean

The correspondence of microstate labeling in EEG and MEG time courses was contrasted against the null distributions. None of the mean Z-scores reached the significance threshold of 3 (see Fig. 9, lower panel). This null result suggests that there is no evidence that MEG microstates co-occur with specific EEG microstates.

**Fig. 10:**
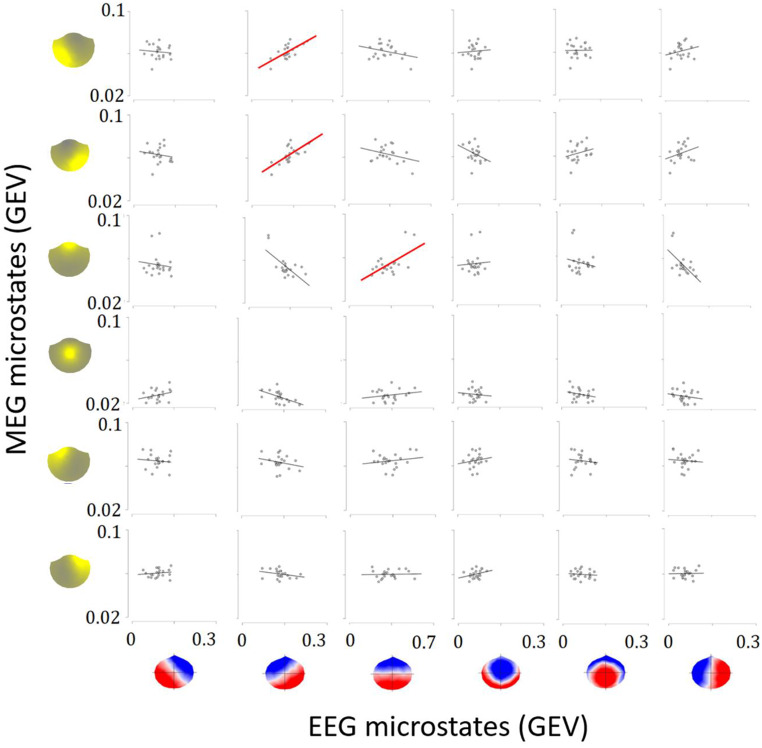
Correlation between GEV of EEG microstates and GEV of MEG microstates. The red lines indicate significant positive correlations

Among all the correlations between the GEVs of EEG and MEG microstates (see Fig. 10), significant positive correlations were found between EEG microstate B and mMS 1 and mMS 2, *r*s > 0.66, *p*s < .001 (FDR corr.) and between EEG microstate C and mMS 3, *r* = .60, *p* = .002 (FDR corr.). All the other correlations were not significant, *p*s > .05.

## Discussion

In this study, we conducted microstate analysis on two distinct MEG datasets. In the first experiment with MEG data recorded during different tasks, we explored the patterns of brain activity labeled by each MEG microstate in the source space, focusing on functional brain networks, and we investigated the functional meaning of MEG microstates by examining their temporal dynamics across different experimental (RCE, ROE, and MMN) and stimulus conditions (standard and deviant auditory stimuli). In the second experiment with simultaneous EEG-MEG recording during a resting-state condition with open eyes, we investigated potential links between MEG and EEG microstates at both the temporal and source levels.

In both experiments, we found that six MEG microstates provided the best clustering for the MEG signal, encompassing approximately 60% of the data. However, microstate labeling was substantially lower in MEG than in EEG data, suggesting that the clustering of MEG microstates might be less effective than EEG microstates in classifying topographies at the scalp level. Despite a visual similarity of topographies for mMS 1–4 and EEG microstates A-D, in Experiment 2, no correspondence was demonstrated between MEG and EEG microstates labeling. Specifically, the likelihood of identifying EEG microstate A in the EEG signal did not increase when the MEG signal showed a topographically similar microstate, specifically mMS 1, or any other microstate. In another EEG-MEG study, Coquelet et al. (2022) found no temporal correlation between the labeling of MEG and EEG data, suggesting that EEG and MEG microstates may capture distinct patterns of brain activity contingent upon signal-specific differences. However, we found significant associations of global explained variances of EEG microstate C with mMS 3 and of EEG microstate B with mMS 2 and mMS 1. Hence, although EEG and MEG microstates cluster the electric and magnetic signal differently depending on signal-specific features, individuals preferentially displaying certain MEG microstates also show a greater proportion of specific EEG microstates.

The lack of a clear correspondence between MEG and EEG source activity and the absence of any temporal correspondence of EEG and MEG microstate labeling likely reflect the different nature of the two signals. Contrary to EEG, where brain potentials reflect opposite polarities relative to a reference, in MEG records the polarity depends on the orientation of the brain sources (Corsi 2023). This setup requires the conversion of native values into positive magnitude activities, which we obtained by computing the square root of the sum of the squares for each gradiometer pair. Moreover, EEG and MEG preferentially measure different neuronal sources: radial and tangential currents in EEG but mostly tangential currents in MEG (Corsi 2023). Lastly, EEG electrodes are placed directly on the participant’s head, but MEG sensors are arranged in a helmet-like device in which the position of the head can vary, influencing the sensor-to-head correspondence. Hence, although microstate patterns can be found in EEG and MEG signals, their association with brain activity is influenced by the characteristics of the signal.

Nevertheless, the successful extraction of microstates from MEG data shows the feasibility of identifying recurrent patterns of brain activity also in the magnetic signal. A previous MEG study by Coquelet et al. (2022) identified four distinct MEG microstates: two anterior lateralized (left and right) and two posterior lateralized (left and right) configurations. The two anterior lateralized microstates resembled the topographies of mMS 5–6, while the two posterior lateralized topographies were congruent with those of mMS 1–2. Despite the similarity of topographies, the variance in the number of MEG microstates between Coquelet et al.‘s (2022) study and the present investigation may be due to differences in clustering methodology (i.e., hierarchical clustering with a fixed number of clusters vs. k-means). Interestingly, as reported in the supplementary materials (S1), setting k = 4 in our group-level clustering analysis, we obtained microstate topographies visually similar to those of Coquelet et al. (2022). This partial replication highlights the reliability of unwrapping MEG signals into reproducible microstates. We recommend a meta-criterion validation to determine the ideal number of microstates.

Source reconstruction revealed distinct brain regions associated with the six different MEG microstates, with a high degree of similarity between reconstructed brain sources in the two experiments. Tait and Zhang (2022) implemented a microstate approach to study the MEG signal but moved from scalp-level to source-level data. In contrast to the approach used by Tait and Zhang (2022), which directly clustered source-level activity, in the present study, we first clustered scalp-level activity into MEG microstates and, subsequently, moved to source-level activity to reconstruct the underlying brain generators. At the source level, the reconstructed activities of mMS 1 and mMS 2 are similar to the left and right Parietal cortical microstates, respectively, while the reconstructed activities of mMS 5 and mMS 6 present similarities to the left and right Sensory-Motor cortical microstates. Notably, activities of mMS 1–2 and mMS 5–6 extended beyond these regions, encompassing occipital and temporal regions, which cortical microstates categorize as separate entities, namely Occipital and Fronto-Temporal. Hence, cortical MEG microstates might be more precise than scalp MEG microstates in identifying activities from functionally different brain areas. The reconstructed sources of microstate mMS 3 resembled the activity of the left and right Temporal Pole and the Orbital cortical microstates. However, no cortical microstate exhibited activity resembling the reconstructed centro-medial activity of mMS4.

In Experiment 2, there was a visual correspondence between MEG and EEG microstates at the source level for mMS 1-2-3 and EEG microstate A-B-C. In previous EEG microstate studies with high-density EEG (Bréchet et al. 2019; Custo et al. 2017), occipital sources clustered here by mMS 1 and mMS 2 were found in the two EEG microstates with an anterior/posterior dipole orientation, namely EEG microstates A and B (see also Pascual-Marqui et al. 2014). However, the additional source of EEG microstate A in the left superior temporal gyrus, reflecting the activity of the auditory network (Britz et al. 2010), in MEG, seems to be captured by mMS 5. The similarity observed in brain source activity between mMS 3 and EEG microstate C is not supported by findings from prior studies that investigated source-reconstructed EEG activity (Bréchet et al. 2019; Custo et al. 2017). Moreover, neither Custo et al. (2017) nor Bréchet et al. (2019) reported unilateral activity along the central sulcus, here reconstructed for mMS 5 and mMS 6. Overall, despite some correspondence for occipital brain sources between mMS 1–2 and EEG microstates A-B, the other EEG and MEG microstates seem to be sensitive to activity in different brain regions. Importantly, sources of EEG microstates seem to be distributed in spatially distant brain areas, encompassing regions in different brain lobes. In contrast, MEG microstates may reflect activity more localized in specific brain areas compared to EEG.

The analysis of activity in parcels corresponding to functional brain networks showed that MEG microstates do not reflect the activity of a single brain network or a specific brain function. For example, mMS 5 and mMS 6 are associated with increased activity in Control, Saliency/Ventral Attention, and Somato-Motor networks, but they showed modulations related to auditory deviance detection. Hence, microstates might be linked to dipole localizations on the scalp rather than directly reflecting a specific functional processing or network. Accordingly, task-related modulations of brain activity resulted in increased detection of those microstates topographically closer to the identified source of the activity and decreased detections of more distal ones. Therefore, an open question for future investigation is to what extent MEG microstates reflect a homology of recurring brain activity patterns with regional specificity or whether they model network-level activity patterns across the scalp with heterogeneous sources, i.e., a localization/distribution question on the functional correlates of transient brain topographies.

MEG microstate showed a sensitivity to both task and stimulus manipulations. Instructing participants to keep their eyes closed during resting state significantly increased the time coverage of mMS 1 and mMS 2. Interestingly, the modulation between resting-state tasks correlated with the increase in alpha activity registered by occipital sensors in ROE and RCE: participants who showed the greatest potentiation of alpha power from ROE to RCE presented the most substantial increment of the time coverage of mMS 1–2 in RCE. Stronger alpha power in RCE than ROE is a common observation in resting-state studies (Berger, 1928) and reflects the degree of inhibition of visual information processing (Hohaia et al. 2022). Consistent with this observation, Croce et al. (2020) reported that EEG microstate B is associated with alpha activity (but see, Seitzman et al. 2017; Zanesco et al. 2020).

Modulations of MEG microstates were also observed in the MMN task, both in the global contrast between ROE and MMN and in the stimulus-locked analysis of microstate labeling in segments of standard and deviant auditory stimuli. Compared to ROE, engaging in a task that required watching a silent cartoon determined modulations of mMS 3 and mMS 4. Bréchet et al. (2019) reported that task performance affects EEG microstates C and D. In the analysis of segments locked to the onset of the auditory stimuli, there is an increment of mMS 6 identifications in deviant than standard stimuli during the time window of deviance detection. The presentation of a deviant stimulus increased the detection rate of mMS 6, mainly in participants with the largest MMN amplitude in the right auditory region. Instead, participants with an MMN lateralization to the left showed increased detection rates for mMS 5. Tait and Zhang (2022) reported increments of the Fronto-Temporal cortical microstate to auditory processing. Hence, microstates extracted during the resting state, as in the present experiment, or during the whole task duration, as in Tait and Zhang (2022), are sensitive to local activity changes when fitted to the activity evoked by a specific stimulus presentation.

In conclusion, six MEG microstates were identified. MEG microstates were sensitive to global and local activity, such as occipital alpha increments during RCE or event-related auditory activity during a passive auditory MMN task. While our findings align with previous MEG studies at both sensor and source levels, their association with EEG microstates remains to be established. Nevertheless, task- and stimulus-specific modulations of MEG microstates indicate that they can detect spontaneous resting-state brain activity and are sensitive to changes in brain activity during a task, offering a tool for studying normal or dysfunctional brain functioning.

## Electronic Supplementary Material

Below is the link to the electronic supplementary material.

Supplementary Material 1

## Data Availability

The data that support the findings of this study are available on request from the corresponding authors.
