# Supplementary material for: MEG Microstates: An Investigation of Underlying Brain Sources and Potential Neurophysiological Processes
Source: Brain Topogr. 2024 Aug 8;37(6):993–1009. doi: 10.1007/s10548-024-01073-z (PMC11408537; doi:10.1007/s10548-024-01073-z)

**SUPPLEMENTARY MATERIALS**

S1 K-MEANS CLUSTERING

The group k-means cluster results for each k number of clusters (from 1 to 20). The meta-criterion validation implemented in Cartool indicated K=6 as the optimal number of clusters. In parentheses, the number of clusters after merging highly correlated cluster maps (threshold set at 80%).

The cluster maps with K=4 were similar to those found in a previous MEG microstate study (Coquelet et al., 2022).


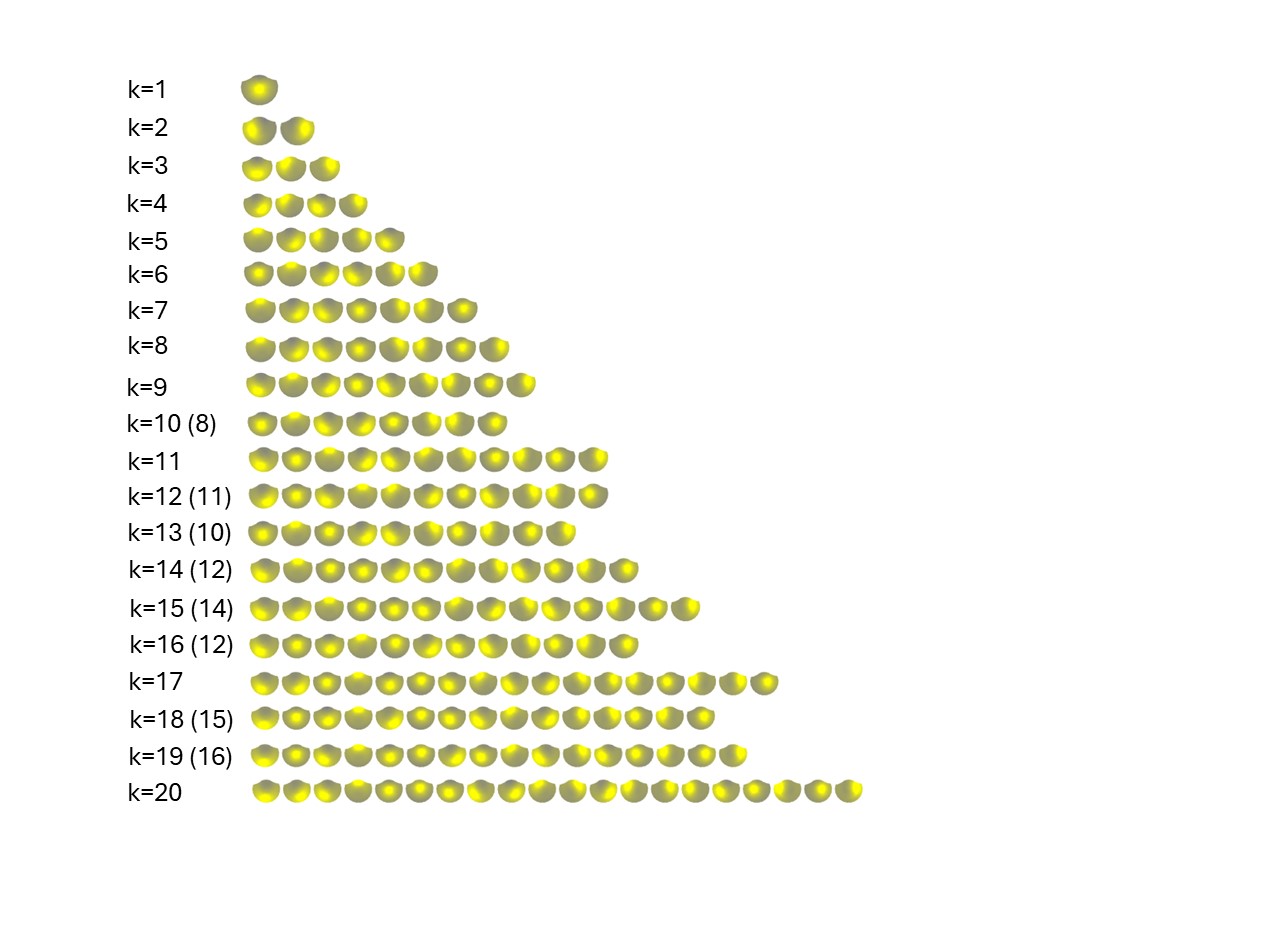

Supplement: Supplementary file 1 — Supplementary Material 1 [file 10548_2024_1073_MOESM1_ESM.docx]
